# Bimetallic PtAu-Decorated SnO_2_ Nanospheres Exhibiting Enhanced Gas Sensitivity for Ppb-Level Acetone Detection

**DOI:** 10.3390/nano14131097

**Published:** 2024-06-26

**Authors:** Xiaofeng Zhu, Pei Cao, Peng Li, Yue Yu, Ruihua Guo, Yongzhen Li, Hui Yang

**Affiliations:** 1 School of Materials Science and Engineering, Zhejiang University, Hangzhou 310027, China; zhuxiaofeng@bjast.ac.cn; 2 Institute for Smart Ageing, Beijing Academy of Science and Technology, Beijing 100089, China; caopei2022@163.com (P.C.); lipeng2023@bjast.ac.cn (P.L.); yuyue@bjast.ac.cn (Y.Y.)

**Keywords:** PtAu bimetal, SnO_2_ nanospheres, acetone, gas sensor

## Abstract

Acetone is a biomarker found in the expired air of patients suffering from diabetes. Therefore, early and accurate detection of its concentration in the breath of such patients is extremely important. We prepared Tin(IV) oxide (SnO_2_) nanospheres via hydrothermal treatment and then decorated them with bimetallic PtAu nanoparticles (NPs) employing the approach of in situ reduction. The topology, elemental composition, as well as crystal structure of the prepared materials were studied via field emission scanning electron microscopy, transmission electron microscopy, X-ray photoelectron spectroscopy, and X-ray diffraction. The findings revealed that bimetallic PtAu-decorated SnO_2_ nanospheres (PtAu/SnO_2_) were effectively synthesized as well as PtAu NPs evenly deposited onto the surface of the SnO_2_ nanospheres. Pure SnO_2_ nanospheres and PtAu/SnO_2_ sensors were prepared, and their acetone gas sensitivity was explored. The findings demonstrated that in comparison to pristine SnO_2_ nanosphere sensors, the sensors based on PtAu/SnO_2_ displayed superior sensitivity to acetone of 0.166–100 ppm at 300 °C, providing a low theoretical limit of detection equal to 158 ppm. Moreover, the PtAu/SnO_2_ sensors showed excellent gas response (R_a_/R_g_ = 492.3 to 100 ppm), along with fast response and recovery (14 s/13 s to 10 ppm), good linearity of correlation, excellent repeatability, long-term stability, and satisfactory selectivity at 300 °C. This improved gas sensitivity was because of the electron sensitization of the Pt NPs, the chemical sensitization of the Au NPs, as well as the synergistic effects of bimetallic PtAu. The PtAu/SnO_2_ sensors have considerable potential for the early diagnosis and screening of diabetes.

## 1. Introduction

Early diagnosis and screening of chronic diseases are important factors for improving cure rates. Concentration changes of volatile organic compounds (VOCs) in human exhalation are closely linked to certain diseases, and analyzing these VOCs is a non-invasive and convenient method [1]. Among different gas biomarkers, acetone is the key factor reflecting the progress of diabetes and/or therapeutic effect, and its concentration can be used as the basis to diagnose diabetes [2]. Therefore, the rapid and effective detection of acetone is highly desirable. According to clinical analyses, the level of acetone exhaled by healthy people is 300–900 ppb, whereas its level exhaled by diabetic patients exceeds 1.8 ppm [3,4]. Faced with extremely low target gas (TG) concentrations and complex background environments, stricter requirements are required to construct high-performance gas sensors, enabling the recognition of acetone in breath at ppb levels with improved selectivity. Chemiresistive-gas-sensor-based non-invasive respiratory diagnoses have become a promising technique for portable disease screening compared to other respiratory component detection technologies due to their superior sensitivity, good material designability, low cost, compact size, easy integration into sensor arrays, and low power consumption [5,6].

Gas sensors employing metal oxide semiconductors (MOSs) are more convenient to carry and operate and respond more rapidly compared to traditional methods [7,8]. The core component of gas sensors, the MOS, offers the benefits of low price, strong specificity, and a facile manufacturing process [9]. MOS materials have a considerably broad application prospect in non-invasive and rapid diagnosis fields. Among them, Tin(IV) oxide (SnO_2_), a common n-type broad bandgap semiconductor (*E_g_* = 3.6 eV at a temperature of 300 K), is extensively employed as a gas sensor material due to its excellent stability as well as improved conductivity [10,11]. However, gas sensors solely relying on SnO_2_ display drawbacks including poor response, limited selectivity, and excessively high operating temperatures (*T*o) [12]. Therefore, to improve its gas sensitivity, researchers have sought to modify SnO_2_ using various strategies, including changing the crystal structure and morphology, decorating catalysts, constructing heterostructures, developing charge transfer hybrids, and incorporating molecular probing along with sieving effects [13,14,15,16,17]. Among these strategies, the use of functionalized nanocatalysts involving hybrid nanocomposites with synergistic effects as well as the introduction of significantly active catalysts for gas chemisorption demonstrates remarkably enhanced sensitivity and selective detection ability [13].

Bimetallic nanoparticles (NPs), a type of heterogeneous catalyst, exhibit dual properties related to their parent metal. By introducing guest metals, the activity, durability, and selectivity of the bimetallic NPs can be considerably adjusted and enhanced due to their reported synergistic effects. This unique advantage provides enormous possibilities for high-performance gas sensors. Researchers such as Li et al. [18] demonstrated that bimetallic PdAu functionalization on SnO_2_ nanosheets achieved the highly sensitive detection of formaldehyde and acetone and exhibited excellent reusability, reliability, and strong resistance to humidity and corresponding biomarkers in the human exhaled air. Zhu et al. [19] prepared PdPt/In_2_O_3_ nanospheres featuring various Pt and Pd ratios, achieving prompt hydrogen detection at 400 ppb, as well as improved selectivity, repeatability, and prolonged stability. The enhanced gas sensitization was because of the synergistic effects of Pd and Pt. Luo et al. [20] developed a ZnO hollow nanocube decorated with PdRh, which had a hollow framework rich in Rh and a Pd-rich core frame. The results of the study revealed that it exhibited improved H_2_S-sensing properties. These enhancements were credited to the outstanding conductivity of its framework, which improved gas diffusion and specific surface area, the excellent catalytic capability of the PdRh bimetal, as well as the establishment of Schottky barrier-type junctions and defects. Feng et al. [21] synthesized AgPt-decorated WO_3_ NPs that exhibited a significantly enhanced response towards acetone. This was because of the electronic as well as the chemical sensitization of the Pt and Ag NPs to the WO_3_ NPs that enhanced the adsorption of oxygen and improved the reaction rate, thereby promoting the gas-sensing response. Therefore, the preparation of SnO_2_ with a unique structure followed by functionalization with bimetallic NPs on the surface emerges as a significant strategy for producing sensing materials characterized by enhanced response levels and rapid response and recovery times, ideal for determining trace-level concentrations of acetone.

This study presents the fabrication of bimetallic PtAu-modified (PtAu/SnO_2_) SnO_2_ nanospheres while employing the simple and versatile approaches of hydrothermal [22,23] and in situ reduction. The structure as well as morphology of the SnO_2_ nanospheres before and after the modification were examined through SEM, TEM, XRD, and XPS. Furthermore, the gas-sensitive-sensing efficiency of the obtained materials was explored by employing acetone as the analyte. The novelty of this study is evident in these two key elements: a simple fabrication approach along with outstanding gas detection efficiency. We prepared SnO_2_ nanospheres by the hydrothermal method with tin tetrachloride (SnCl_4_) as the precursor followed by decorating them with PtAu NPs on the SnO_2_ surface through the in situ reduction of chloroplatinic acid (H_2_PtCl_6_) and chloroauric acid (HAuCl_4_) employing ascorbic acid. The approach is considerably simple as well as efficient and does not introduce any surfactant. PtAu/SnO_2_ displayed enhanced selectivity, sensitivity, and rapid response and recovery times for acetone gas at the optimal response temperature (300 °C). The enhanced sensitivity was because of the electronic and chemical sensitization, as well as the synergistic effect of bimetallic PtAu. In addition, the ppb-level determination of acetone in breath was important to diagnose diabetes. In conclusion, the mechanism of gas detection in PtAu/SnO_2_ nanospheres was deliberated.

## 2. Experimental Section

### 2.1. Materials and Precursors

SnCl_4_ (99%), sucrose (99.5%), H_2_PtCl_6_ (8 wt.% in H_2_O), HAuCl_4_ (98%, Au 47.8%), as well as ascorbic acid (≥99.0%) were supplied by Anhui Zesheng Technology Co., Ltd., Anqing, China. All the reactants employed for the synthesis were of analytical grade and were utilized as provided without any purification. Distilled (DI) water was employed during the entire study.

### 2.2. Preparation of SnO_2_ Nanospheres

SnO_2_ nanospheres were prepared via a straightforward one-step hydrothermal technique followed by calcination treatment. In a typical synthetic strategy, SnO_2_ nanospheres were fabricated by dissolving 1.5 mmol of SnCl_4_ in DI water (15 mL) while continuously being stirred, followed by the addition of 3 mmol sucrose. Following that, the prepared solution was moved to a 50 mL stainless steel autoclave lined with Teflon and kept at 180 °C (12 h). The precipitate was then left to cool to room temperature (RT) followed by centrifuging and washing multiple times with ethanol and DI water. Finally, the SnO_2_ samples underwent drying at a temperature of 80 °C (6 h), followed by calcination in air for 4h at a temperature of 500 °C.

### 2.3. Synthesis of PtAu/SnO_2_ Nanospheres

**Figure 1:**
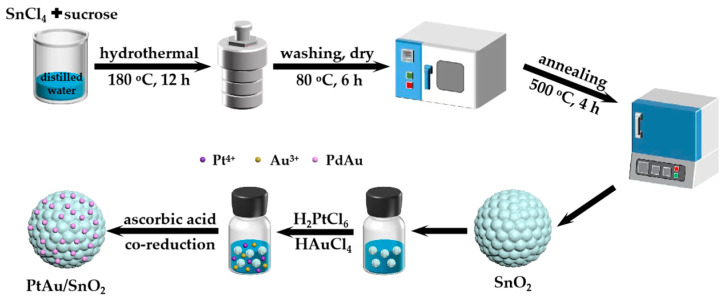
Fabrication of SnO_2_ nanospheres decorated with PtAu NPs.

Figure 1 illustrates the synthesis of SnO_2_ nanospheres decorated with bimetallic PtAu (PtAu/SnO_2_). PtAu/SnO_2_ decoration was carried out through the in situ reduction of H_2_PtCl_6_ along with HAuCl_4_ employing ascorbic acid acting as the reduction agent. The fabrication was carried out by dispersing 0.1 g of SnO_2_ nanospheres in DI water (10 mL) and ultrasound-treated (10 min). Following that, 130 μL HAuCl_4_ (20 mM) and 230 μL H_2_PtCl_6_ (20 mM) were introduced to the obtained mixture. Following a brief interval, 1 mL of ascorbic acid solution with a molarity of 0.1 M was introduced to the mixture followed by stirring (4 h) at RT conditions. At last, the products (PtAu/SnO_2_) were centrifuged and underwent washing using DI water followed by drying at 60 °C. Similarly, the fabrication of Au/SnO_2_ and Pt/SnO_2_ was carried out for comparison employing 130 μL HAuCl_4_ (20 mM) and 230 μL H_2_PtCl_6_ (20 mM), respectively.

### 2.4. Material Characterization

Powder XRD (D/MAX-2600; Rigaku, Tokyo, Japan) was performed through a Cu-Kα radiation source (λ = 1.54056 Å, 150 mA, 40 kV) within the range spanning from 10° to 80°. The prepared samples were studied using a field emission scanning electron microscope (FE-SEM, TESCAN MIRA LMS, TESCAN, Brno, Czech Republic) coupled with an energy-dispersive X-ray spectroscope at 15 kV, as well as an energy-dispersive X-ray (EDX) spectroscope. The TEM and the high-resolution transmission electron microscopy (HRTEM) images (JEM-F200, JEOL, Tokyo, Japan) were acquired through an accelerating voltage of 200 kV. XPS (K-Alpha, Thermo Scientific, Waltham, MA, USA) studies were carried out employing an Al Kα radiation source (1486.6 eV, 50 eV pass energy, 400 μm spot size), and all measured binding energies (BEs) corresponded with the C 1s peak found at 284.6 eV of surface indefinite carbon. Spectra were analyzed using Avantage 5.9931 software (Thermo Scientific). An inductively coupled plasma mass spectrometer (ICP-MS) (Agilent 7800, Santa Clara, CA, USA) was also employed for characterizing the samples. The working parameters of ICP-MS are detailed in Table S1.

### 2.5. Gas Sensitivity Experiments

The gas sensor was prepared as a side-heated structure. First, the prepared materials underwent grounding with a dispersant (DI water) within an agate mortar to produce a paste. The prepared paste was then applied employing a brush onto a ceramic tube (CT) upon which Pt-conducting wires and a couple of Au electrodes had been previously printed. The modified CT was dried at 180 °C for approximately 24 h to eliminate the DI water followed by welding onto a pedestal. Heating wires made of Ni–Cr were then pushed into the CT. The resulting sensors underwent aging at 300 °C (3 days) to enhance the mechanical strength and stability of the sensitive films.

A WS-30B gas sensor system from ZhengZhou Winsen Technology Co., Ltd., Zhengzhou, China. was employed to analyze the overall efficiency of the prepared gas sensor. The optimal temperature was determined by applying different heating voltages. Gas characteristics were assessed employing a stationary state gas distribution approach, wherein a standard gas was introduced inside a sealed chamber (18 L). The response of the gas sensor in this work was characterized using the ratio of the sensor resistance in various gas conditions as S = R_a_/R_g_; here, R_g_ and R_a_ denote the resistances of TG and the sensor in air, respectively. The RT relative humidity (RH) of the air was approximately 20%. The response time was specified as the duration required for the sensor to achieve 90% of the overall resistance change from its primary resistance. Conversely, the recovery time was the reverse. The amount of TG acquired from acetone was calculated using Equation (1) [24]:

(1) $$V_{x}=\frac{V\times C\times M}{22.4\times d\times p}\times 10^{-9}\times \frac{273+T_{r}}{273+T_{b}}$$

where *V_x_* (μL) is the liquid volume, *V* (mL) is the testing chamber volume, *M* (g) denotes the liquid molecular weight, *C* (ppm) is the liquid–vapor concentration, *d* (g/cm^3^) is the liquid specific gravity, *T_b_* (°C) is the test chamber temperature, *T_r_* (°C) is the room temperature, and *p* is the purity of the liquid.

## 3. Results and Discussion

### 3.1. Material Characterisation

**Figure 2:**
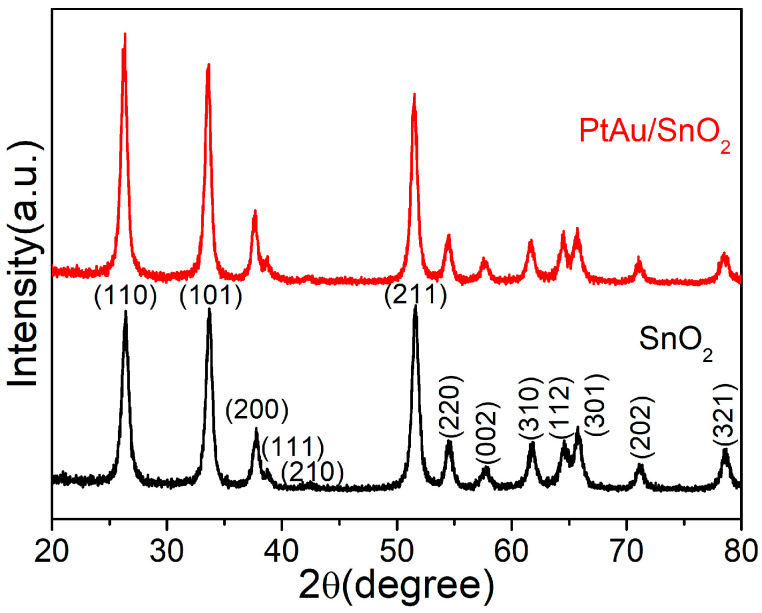
The obtained XRD spectra for the SnO_2_ and PtAu/SnO_2_ nanospheres.

The crystal phases of the pure SnO_2_ nanospheres and PtAu/SnO_2_ nanospheres were analyzed by XRD. It is evident from Figure 2 that all the samples displayed intense diffraction signals and displayed Bragg reflections of the SnO_2_ rutile structure (tetragonal) (110), (101), (200), (111), (210), (211), (220), (002), (310), (112), (301), (202), and (321) planes (JCPDS card no. 41-1445) [25]. No obvious peaks of the metallic NPs (Pt or Au) were found in the PtAu/SnO_2_ nanospheres sample as the limit of detection was not sufficient for the identifying traces of Au and Pt NPs. On the other hand, the presence of Au and Pt was demonstrated by EDX and XPS (see below). The actual Pt and Au content in the PtAu/SnO_2_ nanospheres determined by ICP-MS were 1.39 and 0.43 wt%, respectively.

**Figure 3:**
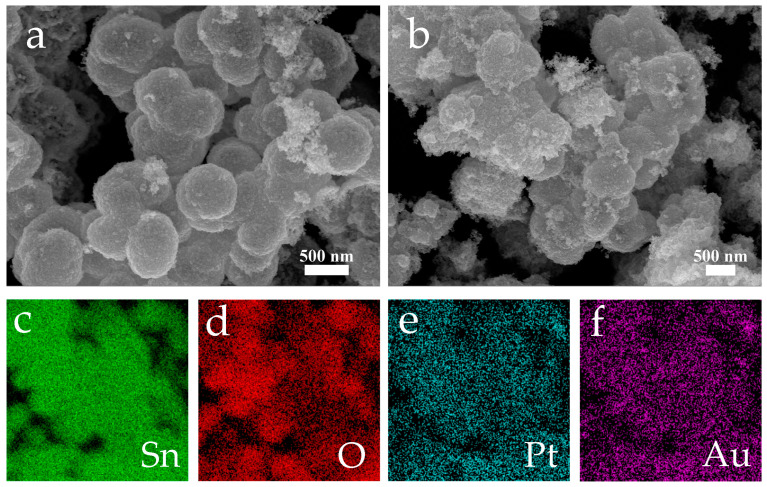
The acquired FE-SEM images for (**a**) the pristine SnO_2_ nanospheres, (**b**) the PtAu/SnO_2,_ and (**c**–**f**) the FE-SEM elemental mapping images of (**b**).

The topologies of the pristine SnO_2_ nanospheres and PtAu/SnO_2_ nanospheres were characterized by FE-SEM. As depicted in Figure 3a,b, SnO_2_ was composed of many accumulative rather than monodispersed nanospheres with diameters of approximately 500 nm. The surface of each individual sphere displayed roughness and comprised densely packed NPs. Elemental analysis by EDX mapping (Figure 3c–f) authenticated the presence of Au, Pt, O, and Sn, traced back to the SnO_2_ nanospheres, while Au and Pt were attributed to the PtAu NPs. In addition, as shown in Figure 3e,f, Pt and Au, respectively, were uniformly distributed on the SnO_2_ nanosphere surface.

**Figure 4:**
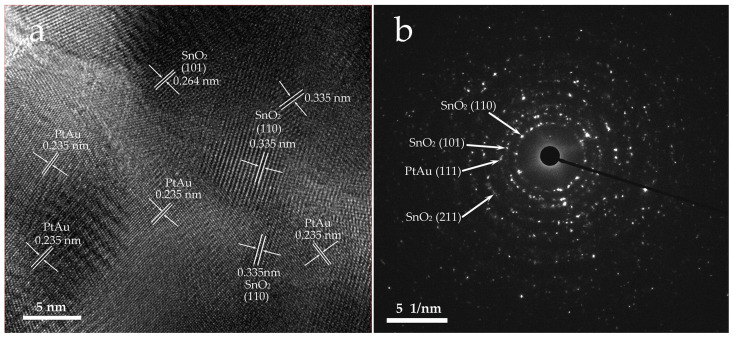
(**a**) The obtained HRTEM image for PtAu/SnO_2_ and (**b**) SAED pattern of the PtAu/SnO_2_.

The microstructure of the functionalized PtAu/SnO_2_ nanospheres was determined by HRTEM. The selected area electron diffraction (SAED) pattern as well as the typical HRTEM lattice image of the PtAu/SnO_2_ are shown in Figure 4. The HRTEM micrograph displayed in Figure 4a shows that the SnO_2_ nanospheres were a single crystal structure with interplanar spacings equal to 0.335 and 0.264 nm, as determined from the lattice fringes that corresponded to the (110) and (101) SnO_2_ rutile structure planes [11]. Furthermore, the characteristic interplanar 0.235 nm spacings correspond to the PtAu (111) planes, suggesting the particle’s structure comprised multiple crystals of both Au and Pt [25,26]. Additionally, the diffraction rings observed in the SAED patterns displayed in Figure 4b were ascribed to the planar directions of (110), (101), (211), and (111), indicating the tetragonal rutile structure of the SnO_2_ crystallites and PtAu NPs.

**Figure 5:**
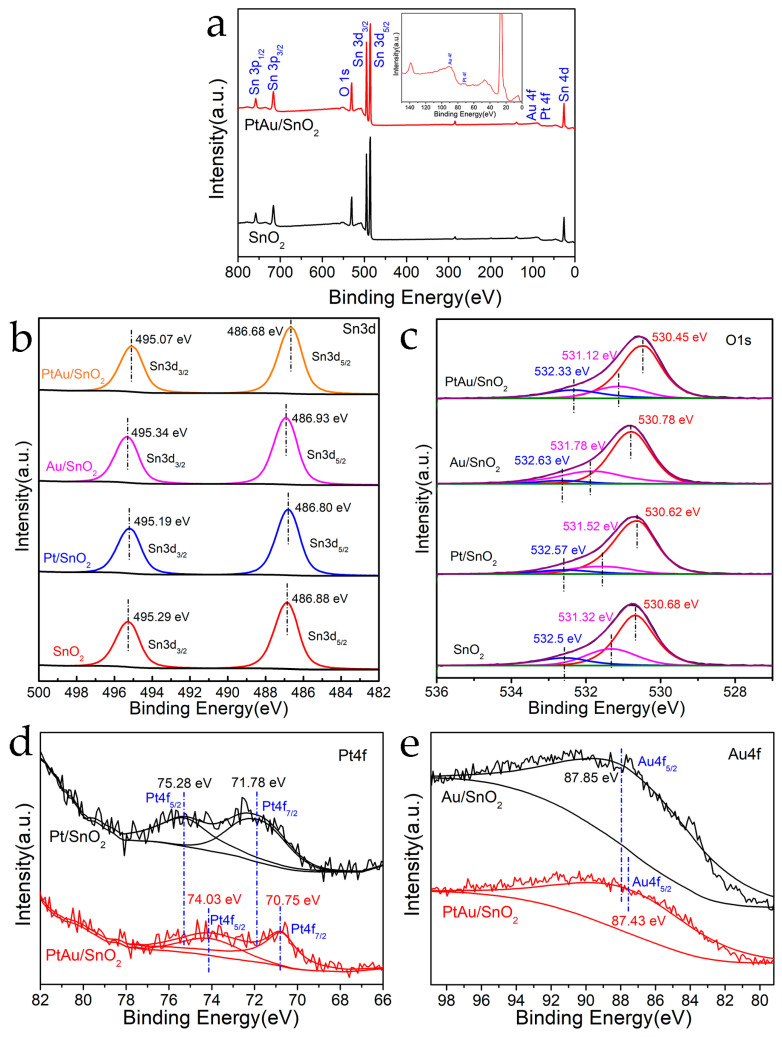
The obtained XPS survey spectrum for (**a**) pristine SnO_2_ and PtAu/SnO_2_, (**b**) Sn 3d, (**c**) O 1s, (**d**) Pt 4f, and (**e**) Au 4f HR-XPS spectrum for pristine SnO_2_, Pt/SnO_2_, Au/SnO_2,_ and PtAu/SnO_2_.

The bonding state and chemical position of the Pt- and Au-loaded SnO_2_ nanospheres were explored through XPS (Figure 5). The survey spectrums for pristine SnO_2_ and PtAu/SnO_2_ are displayed in Figure 5a, confirming the existence of Pt as well as Au in the PtAu/SnO_2_ nanospheres. The high-resolution (HR) XPS analysis of Sn 3d in the pure SnO_2_, Pt/SnO_2_, Au/SnO_2_, and PtAu/SnO_2_ samples is presented in Figure 5b. All materials demonstrated two symmetrical signals, with the BE values derived from Sn 3d_5/2_ and Sn 3d_3/2_ [11,18]. Compared to pristine SnO_2_, because of the incorporation of Au and Pt NPs on the nanosphere surface, the Sn 3d band of the Pt/SnO_2_ and PtAu/SnO_2_ samples moved towards lower BE values, while the Sn 3d signal of the Au/SnO_2_ material shifted towards a higher value of BE. The displacement of the Sn 3d signal was because of the interface of SnO_2_ with the Au as well as Pt NPs, resulting in the change in the electronic structure of the SnO_2_ surface, thereby enhancing the detecting performance. Figure 5c shows the HR-XPS analysis of O 1s in the pure SnO_2_, PtAu/SnO_2,_ Au/SnO_2,_ and Pt/SnO_2_ samples. The O 1s XPS spectrum was resolved into three BE values at 530.45−530.78 eV, 531.12−531.78 eV, and 532.33−532.63 eV, which were associated with lattice oxygen (O_lat_), defect oxygen (O_def_), and adsorbed oxygen (O_ads_), respectively [10]. It is generally believed that the surface O_ads_ ions would facilitate the adsorption process of gas sensing. It was found that the content of O_ads_ in PtAu/SnO_2_ was higher than that of pure SnO_2_, Pt/SnO_2,_ and Au/SnO_2_, indicating that the loading of PtAu NPs could activate and dissociate O_2_ in the ambient air and increase the content of O_ads_, which would participate in the oxidation−reduction reaction, resulting in a larger change in the resistance of PtAu/SnO_2_. The XPS peaks of Pt 4f were found in the obtained samples of Pt/SnO_2_ and PtAu/SnO_2_, as shown in Figure 5d, which were associated with Pt 4f_7/2_ and Pt 4f_5/2_. Similarly, the XPS peaks of Au 4f appeared in the obtained samples of Au/SnO_2_ and PtAu/SnO_2_, as shown in Figure 5e, which were attributed to Au 4f_5/2_. Compared to Pt/SnO_2_ and Au/SnO_2_, the XPS signals of Pt 4f and Au 4f observed in the PtAu/SnO_2_ sample both moved towards lower BE values, further indicating the presence of a combined impact because of modification in the electronic structure of the PtAu NPs.

### 3.2. Gas-Sensing Performance

**Figure 6:**
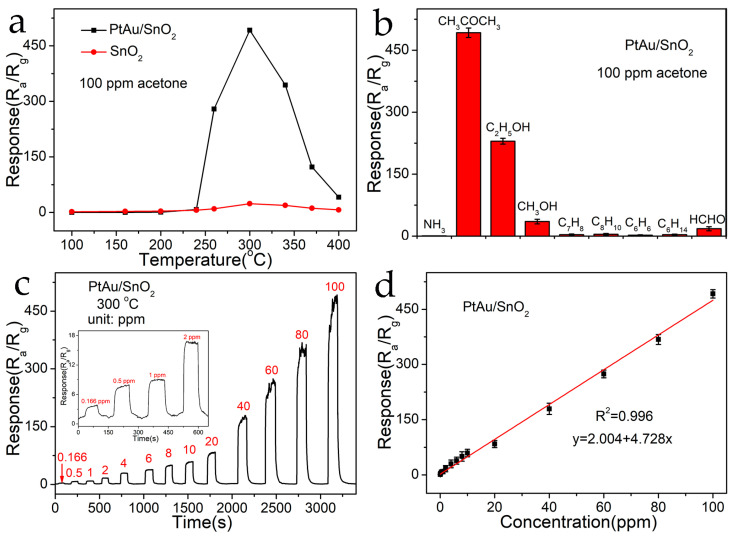
(**a**) Response of pristine SnO_2_ sensors and PtAu/SnO_2_ sensors to 100 ppm acetone at various temperature values. (**b**) Selectivity test of PtAu/SnO_2_ sensors against 100 ppm of NH_3_, CH_3_COCH_3_, C_2_H_5_OH, CH_3_OH, C_7_H_8_, C_8_H_10_, C_6_H_6_, C_6_H_14,_ and HCHO at 300 °C. (**c**) Dynamic response plots of PtAu/SnO_2_ materials to varying amounts of acetone (0.166–100 ppm) at 300 °C. (**d**) Linear response of PtAu/SnO_2_ sensors with acetone gas concentration.

The gas-detecting efficiencies of the pure SnO_2_ as well as the PtAu/SnO_2_ sensors were analyzed and compared. First, the responses of SnO_2_ along with the PtAu/SnO_2_ sensors to acetone (100 ppm) gas were tested in a temperature window spanning from 100 °C to 400 °C to optimize the optimal value To for the sensors (see Figure 6a). The trend of the gas sensitivity response for the sensors first increased to the maximum value as the value of *T*o enhanced, and after that declined with further temperature increased (Figure 6a). As the adsorbed molecular oxygen present on the material surface was activated to produce further active O_2_^−^ and O^−^ species, the response increased as the value of To improved. This effect was observed consistently until the optimum To value was reached. Thereafter, the reaction decreased due to the significant desorption of reactants like reactive oxygen species (ROS) as well as the TG molecules. Given its volatility, the steady-state water of the adsorbed acetone molecules slowly decreased as the temperature enhanced, which led to a lower sensor response. Therefore, the optimized value of To was the equilibrium point between two conflicting mechanisms. It is evident from Figure 6a that the optimized value of To for both the SnO_2_ sensors and PtAu/SnO_2_ sensors was 300 °C. The introduction of the PtAu NPs led to a reduction in the height of the energy hindrance along with an enhancement in activity at the surface, resulting in the much higher response of the PtAu/SnO_2_ sensors to acetone (100 ppm) at 300 °C (R_a_/R_g_ = 492.3) in comparison to the pristine SnO_2_ sensors under the same conditions (R_a_/R_g_ = 23.7). This was associated with the catalytic activity of the Au and Pt NPs, as well as the synergistic effect of the PtAu.

Because the composition of gases exhaled by the human body is complex, it includes a variety of biomarkers. Therefore, the selectivity of the sensor holds importance. The selectivity of the PtAu/SnO_2_ sensors to 100 ppm ammonia (NH_3_), acetone (CH_3_COCH_3_), ethanol (C_2_H_5_OH), methanol (CH_3_OH), toluene (C_7_H_8_), xylene (C_8_H_10_), benzene (C_6_H_6_), n-ethane (C_6_H_14_), and formaldehyde (HCHO) was studied at the optimal To value (300 °C) (Figure 6b). Among them, NH_3_, C_2_H_5_OH, C_7_H_8_, as well as HCHO are typical biomarkers: NH_3_ for kidney disease, C_2_H_5_OH for drunk driving, C_7_H_8_ for cancer, and HCHO for cardiovascular disease. As demonstrated in Figure 6b, the PtAu/SnO_2_ sensors exhibited excellent selectivity for detecting acetone, exhibiting a response sensitivity (R_a_/R_g_) of 492.3, while the response sensitivities (R_a_/R_g_) for NH_3_, C_2_H_5_OH, CH_3_OH, C_7_H_8_, C_8_H_10_, C_6_H_6_, C_6_H_14_, and HCHO under the same conditions were 0.5, 229.8, 35.2, 3.5, 4.3, 2.1, 3.6, and 17.8, respectively.

Figure 6c shows the dynamic gas-sensitive performance of the PtAu/SnO_2_ sensors to various concentrations of acetone (0.166–100 ppm) at the optimal To value of 300 °C. The gas responses of the sensors improved as the acetone concentration improved, and the sensors could be employed for acetone determination across a long concentration range. The results also showed that the proposed sensor displayed an excellent linear correlation with the acetone concentration, with a linear correlation of 0.996 (Figure 6d). Even at trace concentrations of acetone gas, the sensor response was much higher than the measurement noise level. When the PtAu/SnO_2_ sensors were exposed to 166 ppb acetone, the response value R_a_/R_g_ was 3.9. Figure S1 displays the transient response plot of the PtAu/SnO_2_ sensors to 166 ppb acetone at 300 °C. Using the relationship of detection limit (D_L_) as given in Equations (S1) and (S2) for the PtAu/SnO_2_ sensors [10], the D_L_ was 0.158 ppm (158 ppb) (see Supplementary Materials). The actual D_L_ was consistent with the result of the theoretical calculation. This concentration was well below the early diagnosis and monitoring of diabetes breath analysis concentration range: concentrations of acetone in the expired air from patients suffering from diabetes are >1.8 ppm in comparison to 300–900 ppb in the exhaled air from healthy people. Therefore, the PtAu-modified SnO_2_ sensors are sensitive enough to detect the acetone biomarker associated with diabetes.

**Figure 7:**
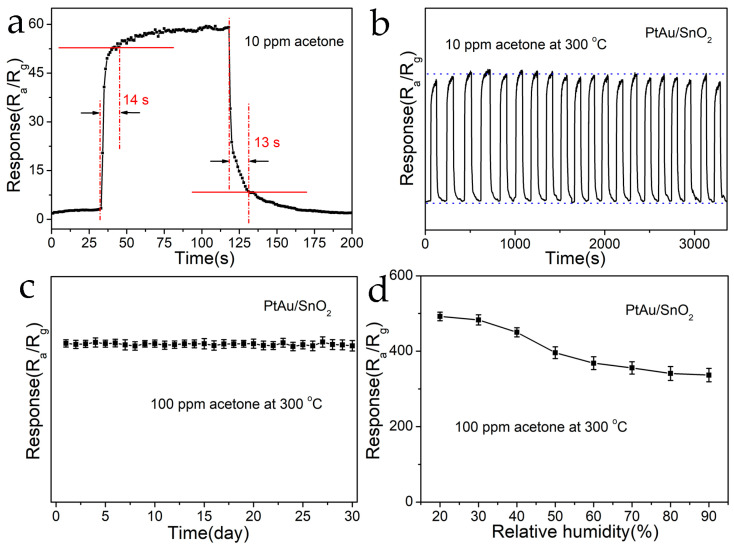
(**a**) The obtained response as well as recovery times for the PtAu/SnO_2_ sensors to acetone (10 ppm) at 300 °C. (**b**) Repeatability of the PtAu/SnO_2_ sensor to acetone (10 ppm) at 300 °C. (**c**) The 30-day stability tests of PtAu/SnO_2_ sensors against acetone (10 ppm) at 300 °C. (**d**) Response of PtAu/SnO_2_ sensor across varying relative humidities to acetone (100 ppm) at 300 °C.

The response along with recovery times were characterized by the durations required for the prepared sensor to achieve 90% of its maximum response and recovery, respectively. These are the primary parameters for gas sensors; rapid and efficient response to TG is an essential characteristic of an outstanding sensor device. The response and recovery times of the PtAu/SnO_2_ sensors to 10 ppm acetone at 300 °C were 14 s and 13 s, respectively (Figure 7a). The sensors revealed an excellent prompt response efficiency. The short time parameter of the PtAu/SnO_2_ sensors was primarily because of the co-catalysis of PtAu, which accelerated the transfer of charge carriers as well as reduced the response and recovery times.

The repeatability of the PtAu/SnO_2_ sensors, required for their practical application, was explored. Figure 7b shows the repeatability curve of the PtAu/SnO_2_ sensors in the presence of acetone (10 ppm) at 300 °C. The sensors underwent evaluation for 20 cycles, and the response values remained nearly consistent in every case, showing no significant variation. They also returned to their initial condition. This demonstrated that the prepared sensors exhibited good recovery and repeatability features. The prolonged stability of the PtAu/SnO_2_ sensors, a further important aspect of their practical use, was also studied. As depicted in Figure 7c, the response of the developed sensor to acetone (100 ppm) at 300 ^o^C was maintained at the original value after 30 days, and the vibration was small, which implied that the gas sensors exhibited good stability over long durations. The high repeatability along with good stability of the PtAu/SnO_2_ sensors were associated with the structural stability of the SnO_2_ nanospheres as well as co-promotion by the PtAu.

Humidity is another important parameter to consider in breath sensing. Figure 7d demonstrates the response curve of the PtAu/SnO_2_ sensor to acetone (100 ppm) at 300 °C in different RH conditions. The results revealed a declining behavior as RH was enhanced in the range of 20–90%, which was possibly due to the reduced interaction between the PtAu/SnO_2_ and acetone gas. Due to the enhanced humidity, additional water molecules got adsorbed onto the PtAu/SnO_2_ surface, which made the chemisorption of oxygen and acetone molecules difficult and prevented the sensing reaction with acetone, thus resulting in decreased sensing performance [27]. When the value of RH reached 90%, the PtAu/SnO_2_ sensor’s response remained at 336.5 (~68.3% of the response at 20% RH), which indicated that the PtAu/SnO_2_ sensors could be used for the detection of acetone at higher humidity.

Table 1 summarizes the comparison of the acetone-detecting characteristics of various gas-detecting materials. The proposed PtAu/SnO_2_ sensors exhibited outstanding acetone gas detecting efficiency. In comparison to published approaches, our newly developed PtAu/SnO_2_ sensor showed higher response values, quick response/recovery times, and low D_L_. The current approach offered a significant strategy for enhancing acetone-sensing performance, particularly in improving response magnitude and reducing response/recovery time.

### 3.3. Gas-Sensing Mechanisms

**Figure 8:**
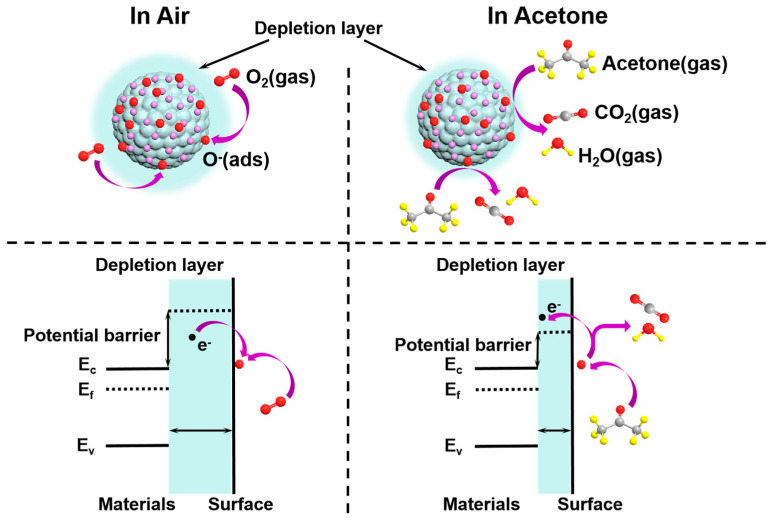
Gas-sensing mechanism of the PtAu/SnO_2_ sensor. The upper images are schematic diagrams, and the bottom images are energy band diagrams.

The sensing mechanism of SnO_2_, a common n-type semiconductor material, follows the surface resistance control model (Figure 8), whereby the resistance variation of SnO_2_ corresponds to the content as well as the type of chemisorbed oxygen (O^−^, O_2_^−^ or O^2−^) during the processes of desorption and adsorption [Equations (2)–(4)]. Typically, when a SnO_2_ sensor operates at an appropriate temperature and comes into contact with air, oxygen molecules adsorb onto its surface. These adsorbed molecules capture electrons from the conduction bands (CBs), forming chemically adsorbed oxygen ions and resulting in an enhanced SnO_2_ resistance [9].

O_2(gas)_ → O_2(ads)_ (2)

O_2(ads)_ + e^−^ → O_2_^−^_(ads)_ (t_op_ < 100 °C) (3)

O_2_^−^_(ads)_ + e^−^ → 2O^−^_(ads)_ (100 °C < t_op_ < 300 °C) (4)

When the SnO_2_ sensor encounters a reducing gas, such as acetone, reactive oxygen ions interact with the TG, releasing electrons into the CBs of the SnO_2_. This leads to a reduction in the resistance value of the SnO_2_ sensor (Figure S2). In the current report, the surface reaction of the SnO_2_ sensor is as follows [Equation (5)] [2]:

CH_3_COCH_3(gas)_ + 8O^−^_(ads)_ → 3CO_2(gas)_ + 3H_2_O_(gas)_ + 8e^−^ (5)

Here, it is evident that the sensor performance depends largely on the content of oxygen ion adsorption. Drawing from the findings of the gas detection characteristics, compared to the simple SnO_2_ sensor, the addition of PtAu can improve the sensing performance of SnO_2_ nanospheres for acetone 20.8 times. The catalytic effect of PtAu improves their sensitivity and selectivity to acetone. This catalytic effect comes from either electronic or chemical sensitization as follows [18,35]. First, Au and Pt NPs are recognized as chemical sensitizers with ‘spillover impacts’. Pt and Au are superior oxygen dissociation catalysts compared to SnO_2_. SnO_2_ has a high availability for catalyzing the breakdown of molecular oxygen, resulting in the generation of ROS that spill over onto the surface of the metal oxides to produce further ROS. The high concentrations of chemically adsorbed O ions lead to a thicker layer where electrons are scarce, which leads to an increase in baseline resistance value within air. As noted in other literature, a thicker depletion zone enhances sensitivity. Therefore, the interaction between the measured acetone gas and chemically adsorbed O ions leads to a substantial change in the value of the resistance. Furthermore, due to the high conductivity and abundance of free electrons in Pt and Au, the adsorption of O ions occurs onto the surface of metal NPs at reduced temperatures (including RT). This is believed to lead to a larger as well as rapid reaction between the determined gas molecules and the adsorbed O. The homogeneous distribution of small Au and Pt NPs on the SnO_2_ nanosphere surface maximizes this impact. Second is the synergistic influence of bimetallic Pt/Au NPs. The synergistic effects in the current study can be interpreted by the following two aspects. First, the introduced PtAu NPs lead to changes in the electronic structure of the SnO_2_ nanospheres at the surface and further activation of the lattice, demonstrated by the shift in the XPS BE band locations. Second, the combined catalytic effect of the PtAu NPs reduces the activation energy, thereby accelerating the reaction course between the acetone molecules and ROS through the uniform distribution of the nanocatalysts.

## 4. Conclusions

Bimetallic PtAu NP–NP-modified SnO_2_ nanospheres were successfully synthesized using the one-step hydrothermal treatment and in situ reduction methods. The prepared PtAu/SnO_2_ nanosphere sensor was applied to the gas-sensitive detection of acetone. Electron microscopy analysis indicated that uniform-sized PtAu NPs are dispersed on the surface of the SnO_2_ nanospheres. The synthetic method was considerably simple and effective and did not introduce any surface activity. This approach can serve as a typical method for modifying bimetallic NPs on the surface of different MOS materials. The PtAu/SnO_2_ nanosphere sensor exhibited a significantly enhanced gas-sensitive response, and it showed good selectivity and sensitivity (R_a_/R_g_ = 492.3), fast response and recovery times (14 and 13 s, respectively), and an acetone gas D_L_ of 158 ppb at the optimal response temperature (300 °C). Compared to pure SnO_2_ nanospheres, modification with bimetallic PtAu NPs was more effective in enhancing the gas-sensitive efficiency of SnO_2_ nanospheres, and their response value was 20.8 times that of pure SnO_2_ nanospheres. This improved gas sensitivity was because of the chemical sensitization of the Au NPs, the electron sensitization of Pt NPs, along with the combined impact of bimetallic. The sensor developed in this study can accurately detect ppb-level acetone gas to meet the needs of the diagnosis and early screening of diabetes. Therefore, PtAu/SnO_2_ nanosphere sensors have considerable potential for the early diagnosis and screening of diabetes.

## Figures and Tables

**Table 1:** A comparison of the acetone recognition efficiency achieved for different materials on the basis of published results and the current work. (Temp. = *T*o, Conc. = acetone concentration, Res./Rec. Time = response/recovery time, *D_L_* = detection limit).

Materials	Morphology	Temp. (°C)	Conc. (ppm)	Res.	Res./Rec. Time (s/s)	*D_L_* (ppb)	Ref.
SnO_2_	Nanosheets	280	1	10.4	40/610	200	[28]
ZnO/SnO_2_	3D inverse opal photonic crystal balls	260	50	40.3	6/10	100	[6]
ZnO/SnO_2_	Thick films	180	0.5	3.36	57/63	10	[29]
Co_3_O_4_/SnO_2_	Yolk-shell nanofibers	350	100	217	0.62/46.5	100	[30]
Zn_2_SnO_4_/SnO_2_	Hierarchical stack structure	275	30	33	13/66	64.25	[31]
Pt_40_Cu_60_/SnO_2_	Octahedral alloy nanocrystal-decorated nanoclusters	240	5	22.04	1.5/58	20	[32]
PdAu/SnO_2_	3D nanosheets	250	2	6.5	5/4	45	[18]
Au/WO_3_-SnO_2_	Corrugated nanofibers	150	0.5	79.6	—	—	[33]
Chitosan-Pt/SnO_2_	Mesoporous nanofibers	350	1	38.4	12/44	5	[34]
PtAu/SnO_2_	Nanospheres	300	10	59.4	14/13	158	Present study

## Data Availability

Data are contained within the article and Supplementary Materials.

## Supplementary Materials

The following supporting information can be downloaded at: https://www.mdpi.com/article/10.3390/nano14131097/s1, Figure S1: operating parameters of ICP-MS; Figure S2: sensing response of the PtAu/SnO_2_ sensors to 1 ppm acetone at 300 °C; Table S1: Operating parameters of ICP-MS; Equation (S1); Equation (S2). Ref. [10] is cited in the supplementary materials.
