# Supplementary material for: Bimetallic PtAu-Decorated SnO2 Nanospheres Exhibiting Enhanced Gas Sensitivity for Ppb-Level Acetone Detection
Source: Nanomaterials (Basel). 2024 Jun 26;14(13):1097. doi: 10.3390/nano14131097 (PMC11243458; doi:10.3390/nano14131097)
Supplement: Supplementary file 1 [file nanomaterials-14-01097-s001.zip › nanomaterials-3057091-supplementary.pdf]

nanomaterials

Supplementary materials

Bimetal PtAu Decorated SnO_2 nanospheres Exhibit Enhanced Gas Sensing Properties for ppb-level Acetone Detection

Xiaofeng Zhu [1,2], Pei Cao [2], Peng Li [2], Yue Yu [2], Ruihua Guo [2,*], Yongzhen Li [2,*], and Hui Yang [1,*]

1 School of Materials Science and Engineering, Zhejiang University, Hangzhou 310027, China; zhuxiaofeng@bjast.ac.cn
2 Institute for Smart Ageing, Beijing Academy of Science and Technology, Beijing 100089, China; caopei2022@163.com (P.C.); lipeng2023@bjast.ac.cn (P.L.); yuyue@bjast.ac.cn (Y.Y.)
* Correspondence: guoruihua@bjast.ac.cn (R.G.); liyongzhen@bjast.ac.cn (Y.L.); yanghui@zju.edu.cn (H.Y.)

Table S1. Operating parameters of ICP-MS

ICP-MS plasma	Parameters
Pump rate	20 r/min
Nebulizer flow	1.00 L/min
Auxiliary gas	1.00 L/min
Sample flush time	40 s
RF power	1550 W

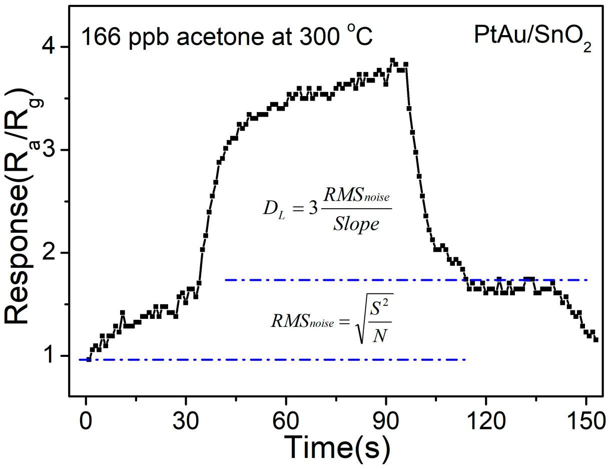

Figure S1. Response transients of the PtAu/SnO_2 sensors to 166 ppb acetone at 300 °C.

The detection limit (D_L) is an important indicator that reflects the sensing performance. D_L is defined as the minimum concentration at which the response differs significantly from the noise signal (typically D_L is 3 times the noise standard deviation) [1]. Sensor noise can be calculated by changing the relative response of the sensor over the baseline. Before exposure to acetone, take the average value of 10 consecutive data points, and calculate the root mean square deviation according to eqn (S1) as 0.2468, where RMS_{noise} is the root mean square error and N is the number of data points. D_L is defined as eqn (S2). According the definition of D_L, for the PtAu/SnO_2 sensors, $D_L = 3 \times 0.2468/4.672 = 0.158$ ppm.

$$RMS_{noise} = \sqrt{\frac{S^2}{N}} \quad (S1)$$

$$D_L = 3\frac{RMS_{noise}}{Slope} \quad (S2)$$

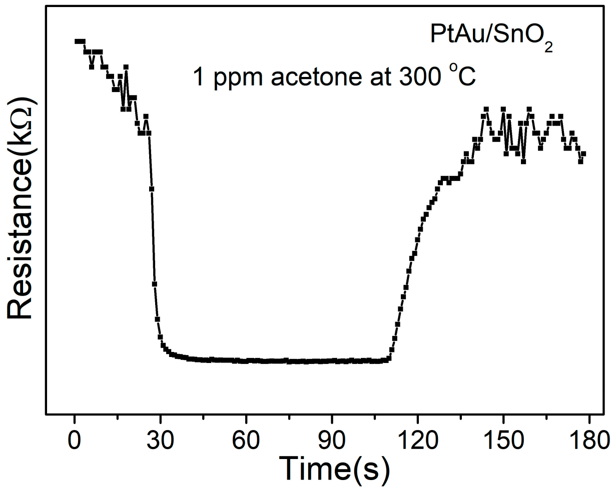

Figure S2. Sensing response of the PtAu/SnO_2 sensors to 1ppm acetone at 300 °C.

References

1. Zhang, X.; Sun, J.; Tang, K.; Wang, H.; Chen, T.; Jiang, K.; Zhou, T.; Quan, H.; Guo, R. Ultralow detection limit and ultrafast response/recovery of the H_2 gas sensor based on Pd–doped rGO/ZnO–SnO_2 from hydrothermal synthesis. *Microsyst. Nanoeng.* **2022**, *8*, 67.
